# Hematological and biochemical reference values of Asian house shrews (*Suncus murinus*) in Bangladesh

**DOI:** 10.14202/vetworld.2019.1514-1518

**Published:** 2019-09

**Authors:** Md. Kaisar Rahman, Shariful Islam, Mizanur Rahman, Jinnat Ferdous, Sazeda Akter, Md. Mustafizur Rahaman, Mohammad Alamgir Hossain, Mohammad Mahmudul Hassan, Ariful Islam

**Affiliations:** 1 EcoHealth Alliance, New York, USA; 2 Institute of Epidemiology, Disease Control and Research (IEDCR), Dhaka, Bangladesh; 3 Department of Pathology and Parasitology, Chattogram Veterinary and Animal Sciences University, Chattogram, Bangladesh; 4 Department of Medicine and Surgery, Chattogram Veterinary and Animal Sciences University, Chattogram, Bangladesh; 5 Bangladesh Forest Department, Ministry of Environment and Forests, Government of the People’s Republic of Bangladesh; 6 Department of Physiology, Biochemistry and Pharmacology, Chattogram Veterinary and Animal Sciences University, Chattogram, Bangladesh

**Keywords:** Asian house shrew, hematology, reference interval, serum chemistry, *Suncus murinus*

## Abstract

**Background and Aim:**

Determining reference values for hematological and biochemical parameters of Asian house shrew (*Suncus murinus*) is important for wildlife research to protect human health in surrounding communities. This study aimed to establish the reference values for selected hematology and serum clinical chemistry analyses that may contribute to research on shrew in future.

**Materials and Methods:**

Blood samples (n=51) were collected from shrews between July and December 2015, Bangladesh, to estimate the levels of hemoglobin (Hb), packed cell volume (PCV), total leukocyte count (TLC), total erythrocyte count (TEC), lymphocyte, monocyte, neutrophil, eosinophil, basophil, calcium, phosphorus (P), sodium (Na), chloride (Cl), urea, glucose, total protein (TP), creatinine, and alanine transaminase (ALT).

**Results:**

Although the values did not differ significantly among sexes, age was found to be a significant factor. Hb, PCV, TEC, glucose, and P were higher in males; eosinophil, Na, Cl, TP, and ALT were higher among females. Adults had significantly greater urea and glucose (p<0.05) while juveniles had insignificantly higher values for TLC, PCV, neutrophil, P, and TP.

**Conclusion:**

This study provides the first reference values for this species in Bangladesh and can be used to guide wildlife research studies.

## Introduction

Hematology and serum chemistry analyses are important indicators in health assessment of domestic animals and wildlife. They are also useful for identifying health disorders, drug toxicity or adverse effects, disease staging, and monitoring of response to treatment. Laboratory animals, especially shrew, rat, and mice, like their wild counterparts, are commonly used for biomedical research. There are 385 species of shrews under 26 genera all over the world. The Asian house shrew (musk shrew) (*Suncus murinus*, Order: Eulipotyphia, Family: *Soricidae*) is native to India and Southeast Asia and widely distributed throughout Bangladesh. The shrew is also natural reservoir of several zoonotic pathogens (Hantavirus, Hepatitis E virus, Thottapalayam virus, Borna disease virus, plague, *Toxoplasma gondii*, etc.) [1-3]. They are found in areas with high human activity. Therefore, they may transfer zoonotic diseases that could present threats to human health [4].

Numerous studies conducted in recent years on the blood parameters of small mammals, particularly rodents, have examined the family *Soricidae*. Variations in the blood parameters of shrew are extremely important in view of ecological and physiological properties of this animal. However, there are no published hematology and serum chemistry data available in Bangladesh or any other region of the Indian subcontinent for this species. Therefore, this study aimed to establish the reference values for selected hematology and serum clinical chemistry analyses that may contribute to research on shrew in the future.

## Materials and Methods

### Ethical approval

The Animal Experimentation Ethics Committee of Chattogram Veterinary and Animal Sciences University (CVASU-AEEC) approved the study protocol and the approval number for the project was CVASU/Dir (R and E) AEEC/2015/07.

### Study sites and subjects

**Figure-1:**
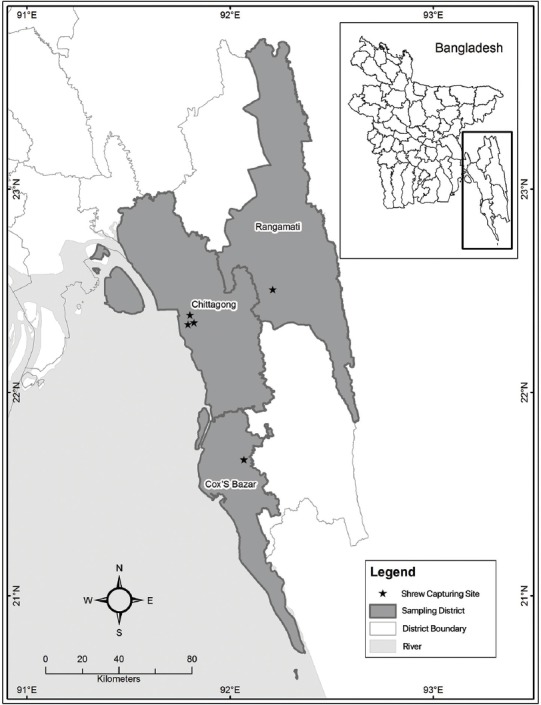
Distribution of Asian house shrews (Suncus murinus) capturing sites in Bangladesh Jul-Dec, 2015.

A total of 51 apparently healthy shrews were captured from Jawtala, Pahartali, and Khulshi of Chattogram, from Chakaria, Cox’s bazar, and from Kaptai, Rangamati (Figure-1) during July-December, 2015. The capturing sites included hilly region, household areas, and agricultural land. Among the captured shrew, 35 (69%) were male and 16 (31%) were female, and 44 (86%) were adults and 7 (14%) were juveniles. Body weight of the animals were ranged from 40 to 195 g. Shrew capture and sample collection were performed following the procedure described elsewhere [5]. Animals were categorized into adult and juvenile based on their appearances described in PREDICT One Health Consortium [6].

### Blood sample collection and analysis

Shrews were captured primarily to identify gastrointestinal parasites after postmortem. After anesthesia, blood samples (1-2 mL/animal) were collected directly from the heart opportunistically. Blood was immediately transferred into two tubes, one without anticoagulant for smooth coagulation and the other with EDTA. After coagulation in the vacutainer, the blood was centrifuged at 3000 rpm for 15 min. Serum samples were obtained and stored in −20°C. The blood samples were analyzed using Hemolyzer 3000 and hematological parameters such as hemoglobin (Hb), erythrocyte sedimentation rate and packed cell volume (PCV), level of calcium (Ca), phosphorus (P), sodium (Na), chloride (Cl), urea, glucose, total protein (TP), creatinine, and alanine aminotransferase were estimated. Red blood cells (total erythrocyte count) and white blood cells were manually estimated and counted [7].

### Statistical analysis

Collected data were recorded in Microsoft Excel 2007 (Microsoft Corporation, Redmond, WA 98052-6399, USA) and imported into MedCalc Statistical Software version 17.5.5 (MedCalc Software bvba, Ostend, Belgium; http://www.medcalc.org; 2017) for estimating mean, standard deviation (SD), and reference intervals (RIs). Robust method was used to calculate RIs because the number of samples collected was 51. The 90% confidence intervals (CIs) were calculated for each RI using bootstrap methods according to Rahman *et al*. [5], Friedrichs *et al*. [8]. Data were presented as mean, SD, minimum, and maximum with 90% CI. Differences between the hematology and serum biochemistry parameters of male and female, adult and juvenile house shrews were analyzed using Student’s t-test or Mann–Whitney test depending on their distribution in histogram. p<0.05 was considered to be statistically significant.

## Results

The results of the blood parameters and serum chemistry analysis (mean, SD, minimum, maximum, and RI – lower limit and upper limit) are given in Table-1. The comparative level of blood parameters and serum chemistry between males and females, adult and juveniles is shown in Table-2. Levels did not vary significantly between males and females. Juveniles showed higher levels of PCV, total leukocyte count (TLC), neutrophils, eosinophils, P, Na, Cl, and TP, whereas adult shrews had higher levels of lymphocyte, urea, and glucose. Among the samples, the mean urea (51.43 mmol/L) and glucose (130.43 mg/dL) levels were significantly higher (p<0.04) in adults compared to juveniles. Elevated mean values were observed for lymphocytes (47.91%) in adults but for PCV (41.83%), neutrophils (45.29%), P (21.35 mg/dL), and TP (9.13 g/dL) in juveniles (Table-2).

**Table 1:** Hematological and biochemical parameters of Asian house shrews (*Suncus murinus*) (n=51) with upper and lower limit of reference interval.

Parameter	Mean	SD	Min.	Max.	RI lower limit (90% CI)	RI upper limit (90% CI)
Hb	13.3	1.1	11.2	15.2	11.3 (10.8-11.7)	15.4 (14.9-15.8)
PCV	39.9	3.3	33.4	45.6	33.39 (32.1-34.7)	46.4 (45.1-47.7)
TLC	7.2	2.3	4.2	11.3	2.80 (1.9-3.7)	11.6 (10.7-12.6)
TEC	4.8	0.9	2.5	7.13	2.91 (2.5-3.3)	6.6 (6.2-6.9)
Lymphocyte	47.2	7.9	29	60	31.65 (28.4-34.9)	62.8 (59.6-66.1)
Neutrophil	42.4	6.9	31	58	28.84 (26.1-31.6)	55.9 (53.2-58.8)
Eosinophil	5.2	2.2	2	11	0.81 (−0.09-1.7)	9.6 (8.7-10.5)
Monocyte	4.8	1.9	2	9	1.13 (0.4-1.9)	8.5 (7.8-9.3)
Basophil	0.3	0.48	0	1	−0.59 (−0.8-−0.4)	1.26 (1.1-1.5)
Calcium	12.3	1.2	8.8	13.9	9.96 (9.5-10.4)	14.6 (14.1-15.0)
Phosphorus	17.5	6.5	10.2	35.7	4.9 (2.3-7.5)	30.2 (27.6-32.7)
Sodium	182.9	21.0	133.2	218.9	141.8 (133.4-150.2)	224.2 (215.7-232.6)
Chloride	136.9	11.3	112.3	161.5	114.7 (110.2-119.3)	159.2 (154.6-163.7)
Urea	50.5	8.1	36.3	67.6	34.7 (31.4-37.9)	66.3 (63.1-69.6)
Glucose	128.4	17.2	97.3	164.9	94.6 (87.7-101.5)	162.1 (155.2-169.1)
TP	7.8	2.8	3.9	13.5	2.2 (1.0-3.4)	13.5 (12.3-14.6)
Creatinine	0.6	0.2	0.3	1.2	0.2 (0.1-0.3)	1.1 (1.0-1.2)
ALT	42.9	7.2	19.7	56.4	28.8 (25.9-31.6)	57.1 (54.1-59.9)

SD=Standard deviation, Min.=Minimum, Max.=Maximum, RI=Reference interval, CI=Confidence interval, Hb=Hemoglobin, PCV=Packed cell volume, TLC=Total leukocyte count, TEC=Total erythrocyte count, TP=Total protein, ALT=Alanine aminotransferase

**Table 2:** Comparison of hematology and biochemistry between clinically healthy male and female, adult and juvenile Asian house shrews (*Suncus murinus*).

Analytes	Male (n=35)			Female (n=16)			p-value	Adult (n=44)			Juvenile (n=7)			p-value
			
	Mean	Std. dev.	Std. err.	Mean	Std. dev.	Std. err.		Mean	Std. dev.	Std. err.	Mean	Std. dev.	Std. err.	
Hb	13.4	0.98	0.16	13.16	1.19	0.29	0.41	13.25	1.06	0.16	13.8	0.9	0.3	0.13
PCV	40.1	3.24	0.55	39.45	3.54	0.89	0.49	39.58	3.30	0.49	41.8	2.8	1.1	0.07
TLC	7.1	2.34	0.39	7.57	2.08	0.52	0.44	6.98	2.15	0.32	8.8	2.4	0.9	0.09
TEC	4.9	0.89	0.15	4.54	1.05	0.26	0.26	4.73	0.91	0.14	4.9	1.2	0.4	0.56
Lymphocyte	47.4	8.25	1.39	47	7.55	1.87	0.79	47.91	7.69	1.16	43.1	9.0	3.4	0.15
Neutrophil	42.5	6.92	1.17	42.31	7.16	1.79	0.97	41.95	6.86	1.03	45.3	7.1	2.7	0.18
Eosinophil	4.9	2.16	0.37	5.75	2.41	0.60	0.31	5.05	2.26	0.34	6.3	1.9	0.7	0.09
Monocyte	4.89	1.88	0.32	4.69	1.9	0.5	0.74	4.82	1.9	0.3	4.9	1.8	0.7	0.93
Basophil	0.34	0.5	0.1	0.3	0.5	0.1	0.83	0.3	0.5	0.07	0.4	0.5	0.2	0.57
Calcium	12.3	1.2	0.2	12.2	1.1	0.3	0.64	12.3	1.1	0.2	12.2	1.7	0.6	0.65
Phosphorus	17.9	7.0	1.2	16.6	5.1	1.3	0.85	16.9	6.0	0.9	21.3	8.2	3.1	0.12
Sodium	181.4	21.9	3.7	186.5	19.2	4.8	0.4	182.2	22.1	3.3	187.9	11.3	4.3	0.51
Chloride	135.9	10.9	1.86	139.2	12.2	3.1	0.4	136.5	11.5	1.7	139.6	10.6	4.0	0.52
Urea	50.7	8.7	1.5	50	6.8	1.7	0.69	51.4	8.01	1.2	44.7	6.3	2.4	0.03*
Glucose	129.8	17.8	3.0	125.2	15.9	3.9	0.39	130.4	16.4	2.5	115.4	18.1	6.2	0.04*
TP	7.5	2.8	0.5	8.5	3.1	0.8	0.28	7.6	2.8	0.4	9.1	3.1	1.2	0.18
Creatinine	0.6	0.2	0.04	0.7	0.3	0.1	0.14	0.7	0.2	0.04	0.7	0.23	0.09	0.45
ALT	42.1	7.9	1.4	44.7	4.9	1.2	0.2	42.8	7.6	1.2	42.84	4.18	1.5	0.99

Std. dev.=Standard deviation, Std. err.=Standard error, Hb=Hemoglobin, PCV=Packed cell volume, TLC=Total leukocyte count, TEC=Total erythrocyte count, TP=Total protein, ALT=Alanine aminotransferase,

* =Significant

## Discussion

Samples were collected from shrews in their natural environment, which is important for captive laboratory animals. In this study, all animals were clinically healthy so the values found were in normal ranges as expected. TLC count was within reference value, but juveniles showed higher TLC values. It was found that hematological values vary along with age and sex in case of *Crocidura russula* [9]. While not found to be statistically significant in this study, Hb and PCV were higher in males than in female shrews, matching similar findings previously reported by Crofton and Share [10]. This is maybe the potential influence of the estrus cycle in female. During proestrus, estrus, and metestrus, the PCV level decreases in female. The percentage of PCV also varies due to age, sea level, altitude, and in some disease situations (e.g., during periods of difficult breathing or dehydration) [11]. Blood glucose concentration was found to be higher in male tree shrews (*Tupaia belangeri chinensis*) in a previous study [12]. Female tree shrews have significantly lower body weight and fasting blood glucose concentration than males (p<0.001) [12]. The concentration of fasting blood glucose was slightly increased with body weight in males; but the body weight, concentration of fasting blood glucose positively increased with age (p<0.001), which is similar to the current study findings. The study revealed that mean urea level is significantly higher in adults (51.43 mmol/L) than in juveniles; this is expected as urea is a primary metabolic product of protein metabolism, which increases with age [13]. Protein quality tests, therefore, should be performed among the same age groups for significant results. No significant variation was found in the level of Na and Cl between adults and juveniles, but both were higher in juveniles than in adults, which support a previous study [14]. TP and creatinine were within the reference range which is relatively similar to the result found in the study of Mock *et al*. [15]. The house shrew showed similar glucose and P level to the least shrew, but the other parameter values were higher in the house shrew [15]. Compared to the previous findings reported for Webster mice, Hb, TP, Ca, and P levels were higher in house shrews in this study [16].

## Conclusion

Although the sample size was small, this study establishes hematological and biochemical reference values for the house shrew to assist in the detection of possible pathologies caused by zoonotically significant organisms. This is the first systematic report of hematology and plasma biochemistry parameters in apparently healthy free-range Asian house shrew and may serve as a reference for clinical assessment and future study of this species.

## Authors’ Contributions

AI initiated and planned the research. SI and MR collected the samples. MR and SA carried out the laboratory work. MKR and JF did a literature review, data analysis, and prepared the draft. SI, MMR, MAH, MMH, and AI made the necessary corrections over the entire manuscript. All authors read and approved the final manuscript.
